# Causal associations between atrial fibrillation and breast cancer: A bidirectional Mendelian randomization analysis

**DOI:** 10.1002/cam4.7067

**Published:** 2024-03-12

**Authors:** Zhaoting Gong, Mengjin Hu, Yuejin Yang, Chunlin Yin

**Affiliations:** ^1^ State Key Laboratory of Cardiovascular Disease, Fuwai Hospital, National Center for Cardiovascular Diseases Chinese Academy of Medical Sciences & Peking Union Medical College Beijing China; ^2^ Department of Cardiology, Xuanwu Hospital Capital Medical University Beijing China

**Keywords:** atrial fibrillation, breast cancer, causal association, Mendelian randomization

## Abstract

**Background:**

Previous observational studies indicated that atrial fibrillation may increase the risk of breast cancer. Following a breast cancer diagnosis, the chance of developing atrial fibrillation may increase as well. However, it is uncertain whether the link is causal or just due to confounding factors.

**Objective:**

Using bidirectional Mendelian randomization (MR) analysis, we sought to assess the bidirectional causal relationship between atrial fibrillation and breast cancer from a genetic level.

**Methods:**

Large genome‐wide association studies yielded summary‐level data for atrial fibrillation and breast cancer. The preliminary estimate was inverse variance weighted (IVW) under a random model. MR–Egger, weighted median, simple mode, weighted mode, and multivariable MR (adjusting body mass index, smoking, and alcohol drinking) were performed as sensitivity analyses.

**Results:**

Genetically predicted atrial fibrillation presented no statistically significant association with overall breast cancer (odds ratio [OR] = 1.00; 95% confidence interval [CI]: 0.97–1.04; *p* = 0.79), estrogen receptor (ER) + (OR = 1.00; 95% CI: 0.96–1.03; *p* = 0.89) or ER− subtypes (OR = 1.00; 95% CI: 0.97–1.04; *p* = 0.89). Similarly, genetically predicted overall breast cancer (OR = 1.01; 95% CI: 0.98–1.04; *p* = 0.37), ER+ (OR = 1.02; 95% CI: 0.99–1.05; *p* = 0.16) or ER− (OR = 0.98; 95% CI: 0.93–1.02; *p* = 0.32) subtypes had no causal effect on atrial fibrillation. Sensitivity analyses yielded similar results. Individual single nucleotide polymorphism had little effect on the total estimate. We did not observe any evidence of horizontal pleiotropy.

**Conclusions:**

Our bidirectional MR studies revealed that there may be no causal links between atrial fibrillation and breast cancer.

## 1 INTRODUCTION

Among the top causes of death worldwide, cardiovascular disease and cancer constitute about nearly half of global deaths.^1^ Atrial fibrillation (AF) is the most prevalent sustained arrhythmia worldwide and a well‐known factor in cardiovascular morbimortality.^2^, ^3^ Besides, AF patients are also confronted with a substantial risk of mortality from non‐cardiovascular causes.^4^ For AF patients receiving oral anticoagulation therapy, over one‐third of deaths are on account of non‐cardiovascular causes, and cancer makes up the greatest proportion of those deaths.^4^ Furthermore, previous study reported that patients with known AF showed a remarkably higher risk of invasive breast cancer. It has also been reported that patients with breast cancer are more inclined to develop cardiovascular diseases,^5^ and AF has emerged as a commonly reported condition among them.^6^ Although they are separate clinical entities, the evidence has shown that the relationship between them seems to be bidirectional. The mutual and reciprocal relationship between these two diseases may be attributed to the convergence of shared risk factors, particularly the enhancement of coagulation‐promoting state and the activation of inflammatory signals.^7^ The development of breast cancer causes inflammation, which is a recognized risk factor for AF, leading to the promotion of the new onset of AF.^8^, ^9^ Furthermore, the use of anti‐cancer treatment such as surgical intervention, radiotherapy, or chemotherapy may potentially predispose to new‐onset AF.^10^, ^11^, ^12^ Nevertheless, the current findings regarding the association between AF and breast cancer are conflicting.^13^, ^14^, ^15^, ^16^, ^17^ Traditional observational studies, however, are subject to the residual confounding effect, overadjustment of potential confounders, and reverse causality,^18^ which may lead to the above‐mentioned conflicting results. It remains unclear whether AF and breast cancer will interact and the bidirectional causal relationship of AF and breast cancer deserves further confirmation.

In general, randomized controlled trial (RCT) is considered the gold standard for establishing causality.^19^ However, due to the sophisticated experimental design, complex implementation process, and rigorous ethical concerns, RCTs are expensive and time‐consuming.^20^ Mendelian randomization (MR) is a method utilizing genetic variants (randomly allocated from parents to offspring) as proxies for the exposure of interest to give insights for causal relationships, preventing bias from confounding factors and reverse causation.^21^ Thus, MR can examine the causality between exposures and outcomes and it has been widely used in the field of cardiovascular diseases and oncology.^22^, ^23^, ^24^, ^25^, ^26^ In the present study, we conducted bidirectional MR analyses to explore a potential causality relationship between AF and breast cancer, which may provide some novel insights and evidence in this field of research.

## 2 METHODS

**FIGURE 1:**
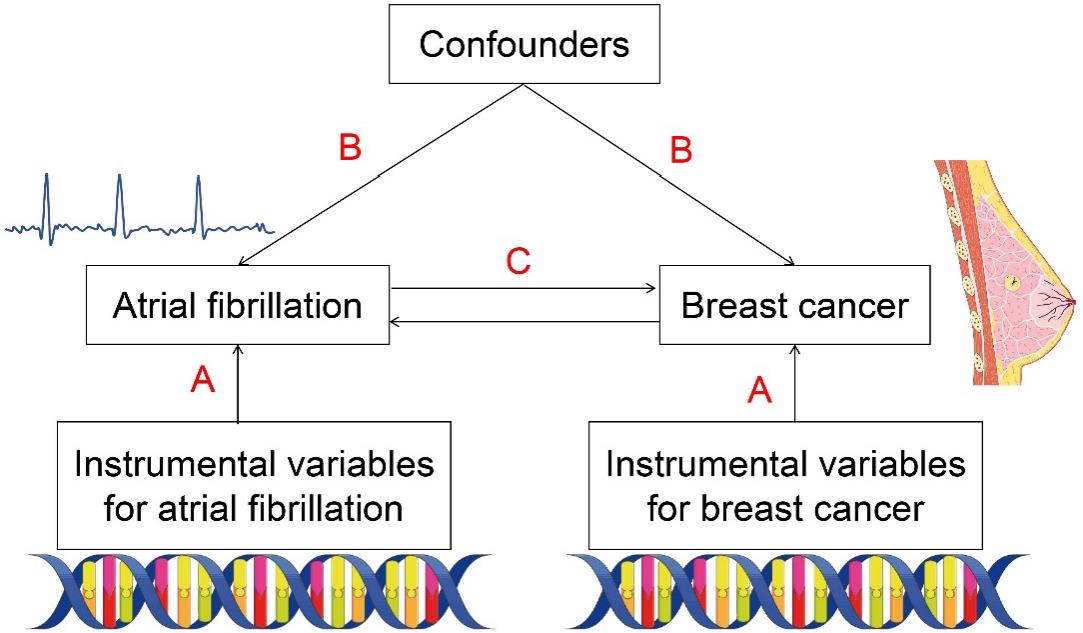
Mendelian randomization model.

Theoretically, MR analysis has to satisfy three assumptions as follows (Figure 1): (A) genetic variants are significantly associated with the exposure of interest (*p* < 5 × 10^−8^); (B) genetic variants are not affected by known confounders of exposure‐outcome associations; (C) there are no other causal pathways connecting the genetic variants to the outcome (directional pleiotropy).

### 2.1 Data sources

Genetic variants associated with AF were obtained from six contributing studies (The Nord–Trøndelag Health Study [HUNT], deCODE, the Michigan Genomics Initiative [MGI], DiscovEHR, UK Biobank, and the AFGen Consortium), which compares a total of 60,620 AF cases and 970,216 controls of European ancestry.^27^ Summary‐level data for overall breast cancer, the estrogen receptor (ER) +, and the ER− subtypes were available from the Breast Cancer Association Consortium (BCAC),^28^ which includes 122,977 breast cancer cases, 69,501 ER+ cases, 21,468 ER− cases, and 105,974 controls (breast cancer free). All cases and controls were females (Table S1). The adjusted variables including body mass index, smoking, and alcohol drinking were obtained from the Genetic Investigation of ANthropometric Traits (GIANT) Consortium,^29^ GWAS and Sequencing Consortium of Alcohol and Nicotine use (GSCAN) Consortium,^30^ and UK Biobank, respectively.

### 2.2 Instrumental variable selection

SNPs that reached genome‐wide significance for the exposures were extracted (*p* < 5 × 10^−8^). Meanwhile, we excluded SNPs with linkage disequilibrium (within a 10,000 kb window, *r* ^2^ > 0.001) to assure statistical independence. These SNPs were then matched and harmonized with the outcome GWAS. To prevent weak instrument bias, we calculated the strength of instrumental variables and deleted SNPs with F‐statistic less than 10.^31^

### 2.3 Statistical analysis

Multiple approaches such as inverse variance‐weighted (IVW), MR–Egger, weighted median, simple mode, and weighted mode were used to assess the bidirectional link between AF and breast cancer. In the absence of directional pleiotropy, the IVW method can provide robust causal estimates.^32^ MR–Egger method allows for directional pleiotropy and the MR–Egger intercept estimates the average pleiotropic effect across all SNPs. If the MR–Egger intercept deviates from zero, directional pleiotropy is present.^33^ Similarly, funnel plots can also identify directional pleiotropy if there is asymmetry. The weighted median method allows some variants to be invalid instruments as long as at least half are valid instruments.^34^ Multivariable MR analysis adjusting body mass index, smoking, and alcohol drinking was also performed. The weighted mode function can provide a reliable estimate provided the most frequent SNP effects are contributed by valid SNP.^35^ Besides, a leave‐one‐out method removing each SNP sequentially was performed to identify outliers that might influence the MR estimates. To assess heterogeneity between individual genetic variants' estimates, we used Cochrane's Q value.^36^ All analyses were carried out with the “TwoSampleMR” package (version 0.5.8) in R version 4.0.3.

## 3 RESULTS

**FIGURE 2:**
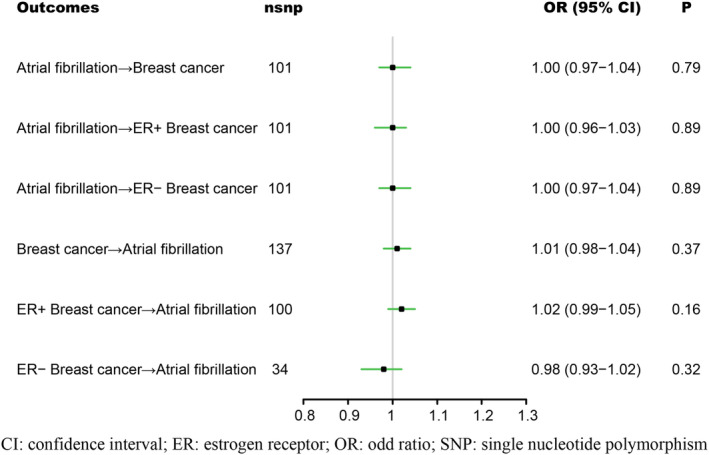
Associations between atrial fibrillation and breast cancer. CI, confidence interval; ER, estrogen receptor; OR, odds ratio; SNP, single nucleotide polymorphism.

According to the IVW results in Figure 2, genetically predicted AF had no causal effect on overall breast cancer (OR = 1.00; 95% CI: 0.97–1.04; *p* = 0.79), ER+ (OR = 1.00; 95% CI: 0.96–1.03; *p* = 0.89) or ER− (OR = 1.00; 95% CI: 0.97–1.04; *p* = 0.89) subtypes. Similarly, genetically predicted overall breast cancer (OR = 1.01; 95% CI: 0.98–1.04; *p* = 0.37), ER+ (OR = 1.02; 95% CI: 0.99–1.05; *p* = 0.16) or ER− (OR = 0.98; 95% CI: 0.93–1.02; *p* = 0.32) subtypes presented no statistically significant association with genetically predicted AF. The multivariable MR analysis adjusting body mass index, smoking, and alcohol drinking also yielded similar results (Table 1). These results were further supported by the weighted median and the MR–Egger methods in Table 1 as well as the simple mode and weighted mode methods in Table S2.

**TABLE 1:** Associations between atrial fibrillation and breast cancer in sensitivity analyses using the weighted median and MR–Egger methods.

Outcomes	Weighted median		MR–Egger		Multivariable MR	
	OR (95% CI)	*p*	OR (95% CI)	*p*	OR (95% CI)	*p*
Atrial fibrillation → Breast cancer	0.99 (0.96–1.03)	0.58	1.03 (0.97–1.09)	0.36	1.01 (0.97–1.05)	0.68
Atrial fibrillation → ER+ Breast cancer	0.98 (0.94–1.03)	0.44	1.02 (0.95–1.09)	0.56	1.01 (0.97–1.05)	0.77
Atrial fibrillation → ER− Breast cancer	0.96 (0.91–1.02)	0.22	1.01 (0.94–1.08)	0.82	0.99 (0.94–1.04)	0.73
Breast cancer → Atrial fibrillation	1.03 (0.99–1.06)	0.15	0.99 (0.93–1.06)	0.74	1.01 (0.97–1.05)	0.66
ER+ Breast cancer → Atrial fibrillation	1.03 (1.00–1.07)	0.08	1.01 (0.95–1.08)	0.70	1.01 (0.97–1.06)	0.49
ER− Breast cancer → Atrial fibrillation	0.98 (0.94–1.03)	0.51	1.00 (0.87–1.15)	>0.99	0.99 (0.95–1.04)	0.65

Abbreviations: CI, confidence interval; ER, estrogen receptor; OR, odd ratio.

**FIGURE 3:**
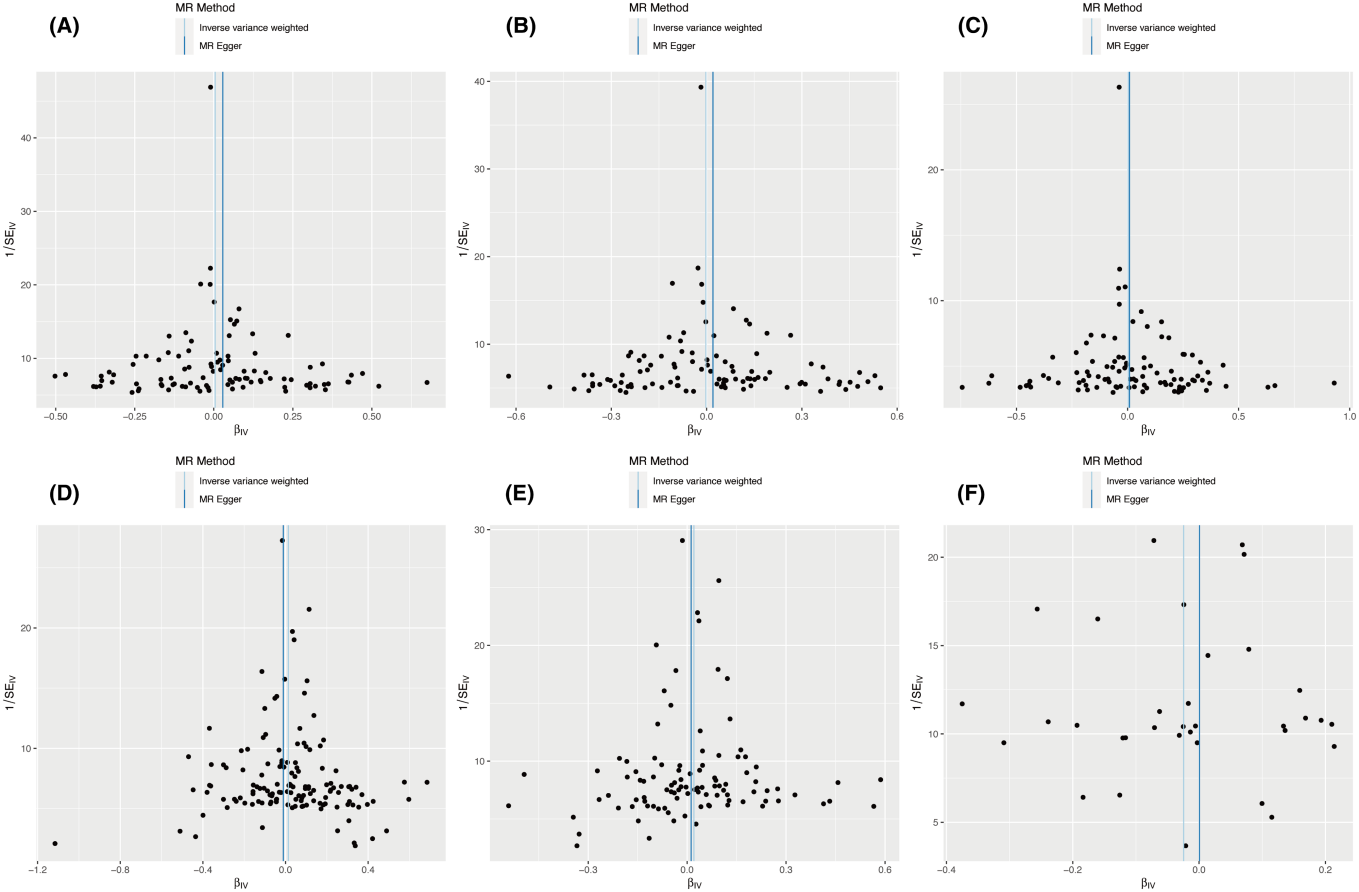
Funnel plot of the associations between atrial fibrillation and breast cancer. A: Atrial fibrillation → Breast cancer; B: Atrial fibrillation → ER+ Breast cancer; C: Atrial fibrillation → ER− Breast cancer; D: Breast cancer → Atrial fibrillation; E: ER+ Breast cancer → Atrial fibrillation; F: ER− Breast cancer → Atrial fibrillation. ER, estrogen receptor.

The scatter plots and forest plots of the associations between AF and breast cancer can be found in Figures S1, S2, respectively. The leave‐one‐out sensitivity analysis revealed that no single SNP disproportionately affected these results (Figure S3). The MR–Egger intercept in Table 2 and funnel plots in Figure 3 revealed no evidence of directional pleiotropy. As there was strong evidence of heterogeneity across SNPs (Table 2), IVW under a multiplicative random effect model was adopted to mitigate the influence of heterogeneity.

**TABLE 2:** Analyses of horizontal pleiotropy and heterogeneity between atrial fibrillation and breast cancer.

Outcomes	Horizontal pleiotropy		Heterogeneity	
	Intercept	*p*	Q	*p*
Atrial fibrillation → Breast cancer	−0.0022	0.37	307	<0.01
Atrial fibrillation → ER+ Breast cancer	−0.0021	0.44	210	<0.01
Atrial fibrillation → ER− Breast cancer	−0.0006	0.85	101	<0.01
Breast cancer → Atrial fibrillation	0.0019	0.41	283	<0.01
ER+ Breast cancer → Atrial fibrillation	0.0008	0.77	239	<0.01
ER− Breast cancer → Atrial fibrillation	−0.0028	0.70	123	0.06

Abbreviation: ER, estrogen receptor.

## 4 DISCUSSION

In the present MR analysis, no causal associations were observed between AF and breast cancer, this suggests that AF diagnosis does not cause an increased risk of breast cancer, and similarly, breast cancer does not cause an increased risk of atrial fibrillation.

Owing to lengthened life expectancy in the general population, cancer and AF both have increasing morbidity, and the coexistence of these two clinical entities has become increasingly pervasive. The coexistence of two diseases has been hypothesized to result from several possible conditions. First, breast cancer and AF share common risk factors including age, obesity, alcohol consumption, and smoking.^3^, ^37^ Second, persistent inflammation is linked to the emergence of breast cancer as well as AF.^38^, ^39^ Third, the occurrence of new AF may be associated with carcinoma‐related therapy such as surgical procedures and chemotherapy.^40^ Both two diseases have an elevated risk of thrombotic, bleeding, and mortality. It has been reported that patients diagnosed with both cancer and AF had twice the risk of thromboembolism and 6‐fold risk of heart failure.^40^ Moreover, the high morbidity of AF in breast cancer patients leads to an increase in cardiovascular mortality.^13^ Therefore, if the relationship between the two is established, AF can be an important comorbidity of cancer patients who require early screening. Vice versa, AF patients also need to pay attention to possible cancer‐related syndrome. The use of cardiovascular medications such as Betablockers can reduce the incidence of AF in patients with breast cancer,^13^ so early application of cardiovascular drugs may play a role in reducing cardiovascular mortality in breast cancer patients. Besides, the utilization of glycosides also leads to a debilitation of the incidence of breast cancer in AF patients,^17^ suggesting that certain agents for the treatment of AF could be a substantial therapy for breast cancer.

The results of the current MR analysis contradicted the results of multiple prior cohort studies indicating an elevated risk of AF in patients with breast cancer.^13^, ^14^ Saliba et al. reported that there was a higher chance of developing AF within the initial 3 months following the diagnosis of breast cancer, but this risk did not persist afterward.^16^ Yun et al. found that the influence of cancer on AF occurrence diminished over time following the diagnosis of cancer. The occurrence of AF within 90 days (HR = 1.48; 95% CI 1.39–1.58) and 1 year (HR = 1.40; 95% CI 1.30–1.50) after being diagnosed with breast cancer was significantly higher. Nevertheless, this association loses significance after five years of cancer diagnosis (HR = 1.00; 95% CI: 0.84–1.18).^40^ Another cohort study revealed that individuals diagnosed with early breast cancer experience a two‐fold higher risk of AF within the initial year after cancer diagnosis. However, they also reported a slight but significant rise in AF incidence 5 years after cancer diagnosis.^14^

Therefore, it is perplexing whether breast cancer is associated with an increased incidence of AF. An increased short‐term risk of new‐onset AF in breast cancer patients was observed in several studies, which can be explained by detection bias since cancer patients might have more medical encounters, and the acute transient state after cancer diagnosis caused by invasive diagnostic measures as well as medical or surgical treatment might also be responsible for this association.^41^, ^42^, ^43^ For the long‐term risk of AF after cancer diagnosis, the conclusions of above observational studies were controversial. Residual confounding inevitably brought by measurement error and incomplete capture of all the confounding factors in the observational study may be one possible cause for the contradictory results.

Additionally, numerous reports have indicated an increased risk of developing cancer after being diagnosed with AF.^44^ However, research results about AF as a potential risk factor for breast cancer were also conflictive. Wassertheil‐Smoller and colleagues found patients with baseline AF had a significantly higher prevalence of invasive breast cancer during a 15 years follow‐up (HR = 1.19, 95% CI: 1.03–1.38).^17^ A registration study of all Danish patients found a five‐fold increase in the risk of cancer diagnosis in patients with AF within the first three months after AF diagnosis. Furthermore, the standard incidence rate (SIR) of breast cancer in patients with AF was 3.89 (95% CI: 3.50–4.30) within the first three months after AF diagnosis, while the SIR after the initial 3 months was 1.16 (95% CI: 1.11–1.21).^44^ Another analysis showed that risk of breast cancer increased in the first 90 days after AF diagnosis, while the risk of breast cancer was significantly reduced after the first 90 days.^16^

Multiple studies have indicated that the likelihood of developing breast cancer was notably higher within a 3‐month period following the diagnosis of AF. It is possible that this association is influenced by detection bias, as increased healthcare interactions and bleeding resulting from anticoagulation therapy after AF diagnosis could reveal previously hidden malignant tumors. Moreover, it was reported that cancer cases were more likely to metastasize when diagnosed, which may indicate that AF is less likely to cause cancer but more likely to be a potential biomarker for occult cancer.^44^, ^45^ Considering that the studies discussed above are all observational studies, the lack of control over residual confounding may be an important explanation for the contradictory results about the long‐term risks of breast cancer after AF diagnosis.

Besides, our results showed that the causal link between breast cancer subtypes (ER+/ER–) and AF also may not exist. Estrogen plays a crucial role in the growth and development of estrogen‐dependent breast cancer.^46^ Meanwhile, endogenous estrogen and estrogen receptors can directly impact the electrical function of heart.^47^ Therefore, there might be a difference in the incidence of AF among patients with different subtypes of breast cancer. Previous observational studies have shown that patients who did not receive hormonal therapy had a higher risk of AF compared with those who received treatment with hormonal therapy,^13^ while another study reported that estrogen monotherapy seemed to be related to a higher risk of AF.^48^ Nevertheless, research focus on the association between subtypes of breast cancer and AF is limited. More relevant research and high‐level evidence are needed in the future to fill the gap in this field.

Due to the independent selection of the instrumental variable risk alleles without confounding factors, MR analyses are well suited to overcome confounding by unmeasured/unknown factors. Therefore, it is likely that there is no causal relationship between AF and breast cancer. The association reported in previous epidemiological studies may be due to common risk factors, inflammatory reactions, and unidentified residual confounders.

## 5 LIMITATIONS

When interpreting our findings, it is important to assess several limitations of this study. First, the SNP estimates were limited to individuals of European ancestry in order to minimize the potential bias of population stratification, which may affect the generalizability of our findings. Further research is necessary to determine if these findings can be applied to populations from other ethnic backgrounds. Second, we observed evidence of heterogeneity for some outcomes, leading us to adopt a multiplicative random effect model to alleviate the impact of this heterogeneity. Besides, sensitivity analyses apart from the IVW method were performed and similar results were observed, which indicated that our findings were not biased as a result of heterogeneity. Third, as the analysis was based on summary‐level data, individual‐level data such as age, cancer treatments, and cardiovascular comorbidities were not available, which restrained us from further analysis. However, as genetic variants are randomly allocated from parents to offspring, the bias from confounding factors may not influence our results. Fourth, the power of the present analysis is low, which may be explained by the limited number of samples. Further researches are required to validate or refute our findings.

## 6 CONCLUSIONS

The present bidirectional MR studies revealed that the causal links between AF and breast cancer or its subtypes may not exist.

## AUTHOR CONTRIBUTIONS

**Zhaoting Gong:** Conceptualization (lead); data curation (lead); formal analysis (lead); methodology (lead); visualization (lead); writing – original draft (lead). **Mengjin Hu:** Conceptualization (lead); data curation (lead); formal analysis (lead); methodology (lead); writing – original draft (lead). **Yuejin Yang:** Conceptualization (equal); funding acquisition (lead); investigation (equal); project administration (lead); resources (supporting); writing – review and editing (lead). **Chunlin Yin:** Conceptualization (supporting); methodology (supporting); project administration (supporting); writing – review and editing (lead).

## FUNDING INFORMATION

This work was supported by the National Key Research and Development Program of China (2017YFC1700503), CAMS Innovation Fund for Medical Sciences (2016‐I2M‐1‐009), and the National Science and Technology Program during the Twelfth Five‐year Plan Period (2011BAI11B02).

## CONFLICT OF INTEREST STATEMENT

All authors declared no conflicts of interest.

## ETHICS STATEMENT

Written informed consent and ethics approval were not applicable to these analyses because all included genome‐wide association studies (GWAS) data were publicly available and had been approved by the corresponding ethical review board in the original GWAS.

## Supporting information


**Data S1:** Supporting Information.

## Data Availability

The data that support the findings of this study are available from the corresponding author upon reasonable request.
