# Supplementary material for: Causal associations between atrial fibrillation and breast cancer: A bidirectional Mendelian randomization analysis
Source: Cancer Med. 2024 Mar 12;13(5):e7067. doi: 10.1002/cam4.7067 (PMC10928448; doi:10.1002/cam4.7067)
Supplement: Supplementary file 1 — Data S1: Supporting Information. [file CAM4-13-e7067-s001.docx]

**Causal Associations Between** **Atrial Fibrillation and Breast Cancer: A Bidirectional Mendelian Randomization Analysis**

**Running Title:** Atrial Fibrillation and Breast Cancer

Zhaoting Gong, MD^a#^, Mengjin Hu, MD, PhD^b#^, Yuejin Yang, MD, PhD^a,^ *, Chunlin Yin, MD, PhD^b,^ *

^a^State Key Laboratory of Cardiovascular Disease, Fuwai Hospital, National Center for Cardiovascular Diseases, Chinese Academy of Medical Sciences & Peking Union Medical College, Beijing 100037, China

^b^Department of Cardiology, Xuanwu Hospital, Capital Medical University, Beijing, 100053, China.

# Zhaoting Gong and Mengjin Hu contributed equally to this work.

***Corresponding Author**:

Yuejin Yang. Tel: (86)13701151408. E–mail address: yangyjfw@126.com. State Key Laboratory of Cardiovascular Disease, Fuwai Hospital, National Center for Cardiovascular Diseases, Chinese Academy of Medical Sciences & Peking Union Medical College, Beijing 100037, China

Chunlin Yin. Tel: (86)13552566227. E–mail address: yinclmail@163.com. Department of Cardiology, Xuanwu Hospital, Capital Medical University, Beijing, 100053, China.

**Supplementary Figures and Tables**

**Supplementary Figure 1. Scatter plot of the associations between atrial fibrillation and breast cancer**

**
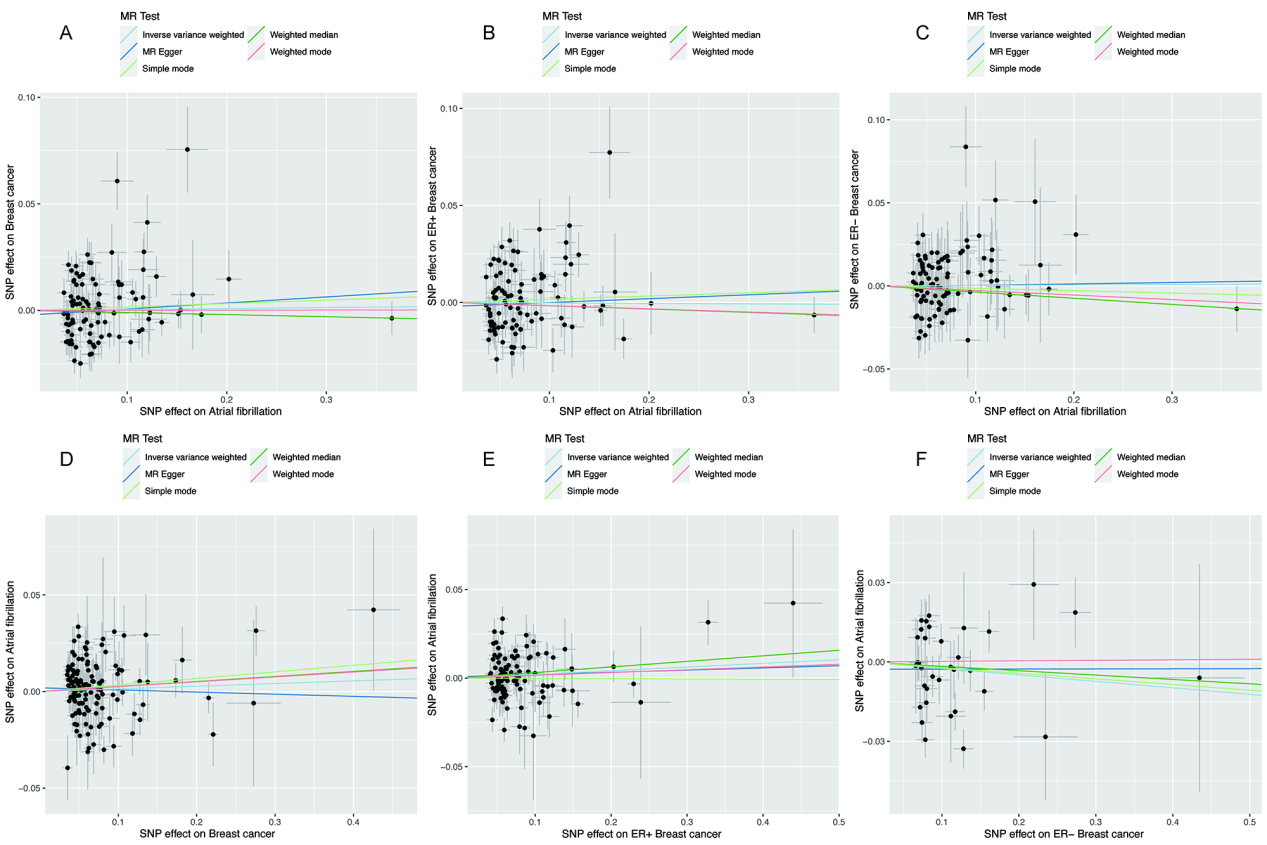
**

A: Atrial fibrillation→Breast cancer; B: Atrial fibrillation→ER+ Breast cancer; C: Atrial fibrillation→ER− Breast cancer; D: Breast cancer→Atrial fibrillation; E: ER+ Breast cancer→Atrial fibrillation; F: ER− Breast cancer→Atrial fibrillation

ER: estrogen receptor; SNP: single nucleotide polymorphism

**Supplementary Figure 2. Forest plot of the associations between atrial fibrillation and breast cancer**

**
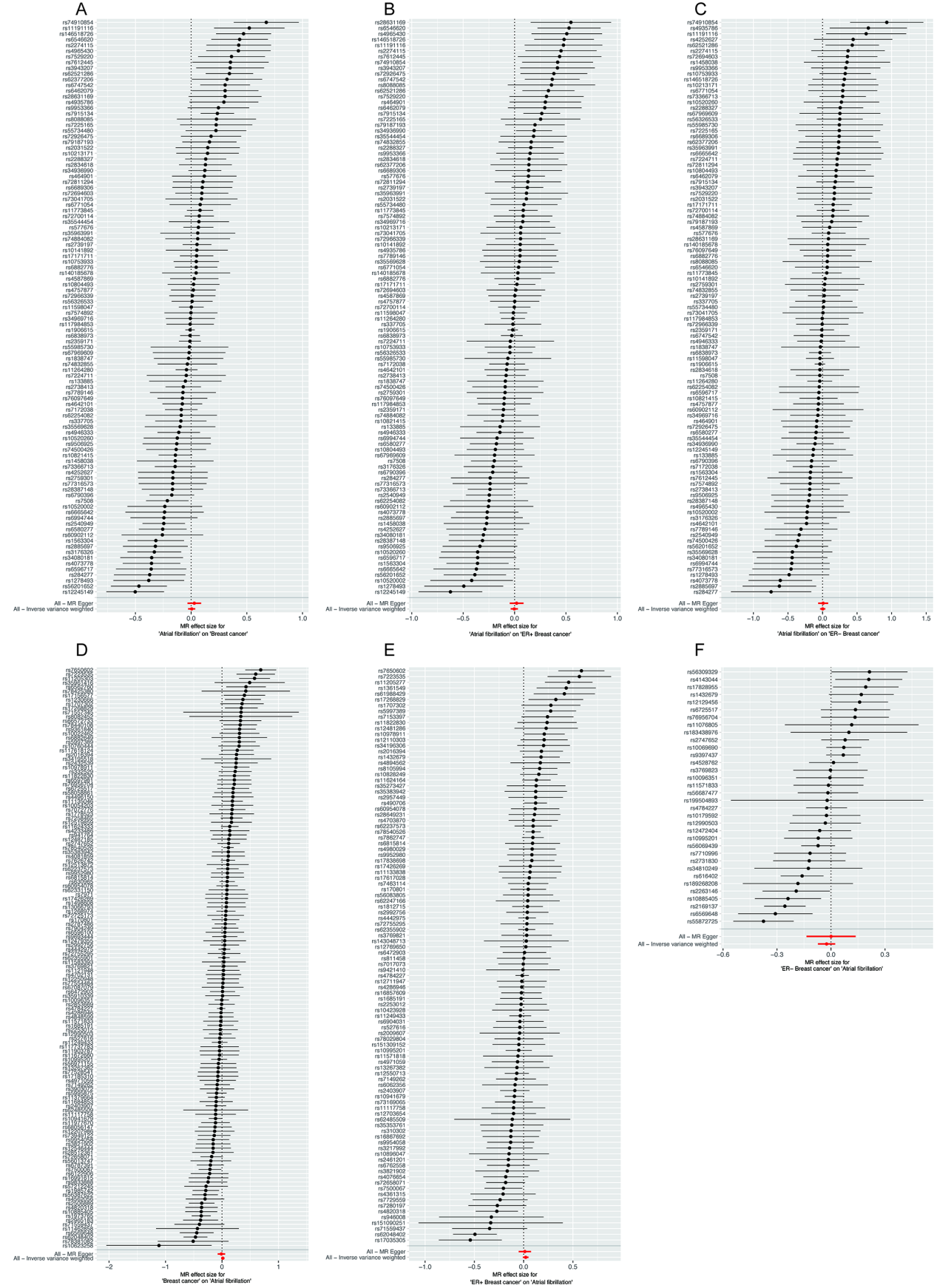
**

A: Atrial fibrillation→Breast cancer; B: Atrial fibrillation→ER+ Breast cancer; C: Atrial fibrillation→ER− Breast cancer; D: Breast cancer→Atrial fibrillation; E: ER+ Breast cancer→Atrial fibrillation; F: ER− Breast cancer→Atrial fibrillation

ER: estrogen receptor

**Supplementary Figure 3. Leave-one-out sensitivity analysis of the associations between atrial fibrillation and breast cancer**

**
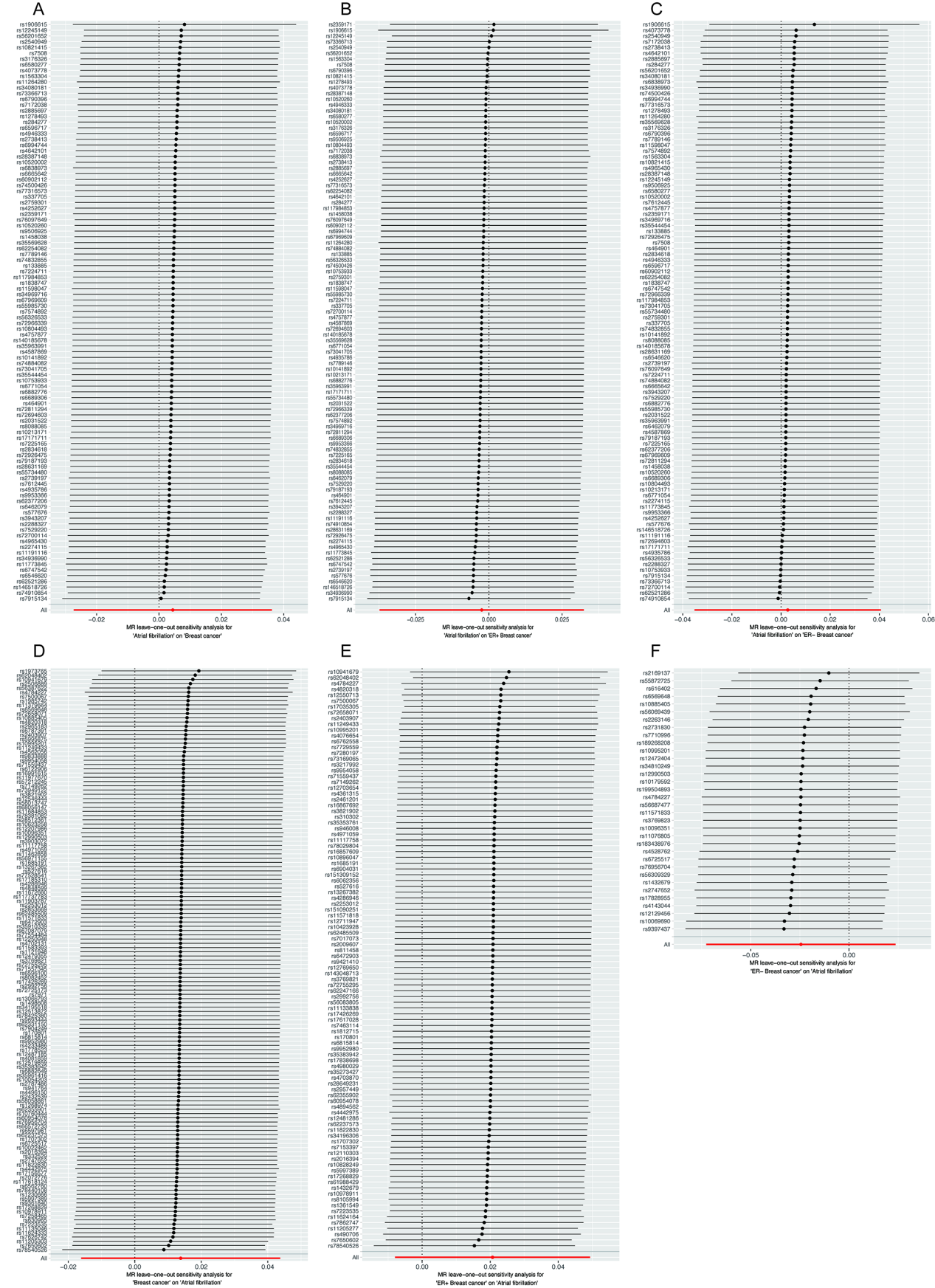
**

A: Atrial fibrillation→Breast cancer; B: Atrial fibrillation→ER+ Breast cancer; C: Atrial fibrillation→ER− Breast cancer; D: Breast cancer→Atrial fibrillation; E: ER+ Breast cancer→Atrial fibrillation; F: ER− Breast cancer→Atrial fibrillation

ER: estrogen receptor

**Supplementary Table 1. Baseline characteristics of included Genome-Wide Association Studies**

| Trait | Year | Author/Consortium | Population | Sample Size | n case | n control | n SNP |
| --- | --- | --- | --- | --- | --- | --- | --- |
| Atrial fibrillation | 2018 | Nielsen JB | European | 1,030,836 | 60,620 | 970,216 | 33,519,037 |
| Breast cancer | 2017 | BCAC | European | 228,951 | 122,977 | 105,974 | 10,680,257 |
| ER+ Breast cancer | 2017 | BCAC | European | 175,475 | 69,501 | 105,974 | 10,680,257 |
| ER− Breast cancer | 2017 | BCAC | European | 127,442 | 21,468 | 105,974 | 10,680,257 |

BCAC: Breast Cancer Association Consortium; ER: estrogen receptor; SNP: single nucleotide polymorphism

**Supplementary Table 2. Associations between atrial fibrillation and breast cancer in sensitivity analyses using the simple mode and weighted mode methods**

| Outcomes | Simple mode | | Weighted mode | |
| --- | --- | --- | --- | --- |
|  | OR (95% CI) | P | OR (95% CI) | P |
| Atrial fibrillation→Breast cancer | 1.02 (0.95-1.09) | 0.65 | 1.00 (0.97-1.03) | 0.97 |
| Atrial fibrillation→ER+ Breast cancer | 1.02 (0.93-1.11) | 0.72 | 0.98 (0.95-1.02) | 0.40 |
| Atrial fibrillation→ER− Breast cancer | 0.99 (0.87-1.11) | 0.82 | 0.97 (0.92-1.03) | 0.36 |
| Breast cancer→Atrial fibrillation | 1.03 (0.96-1.12) | 0.41 | 1.03 (0.98-1.08) | 0.31 |
| ER+ Breast cancer→Atrial fibrillation | 1.00 (0.92-1.08) | 0.97 | 1.02 (0.97-1.06) | 0.52 |
| ER− Breast cancer→Atrial fibrillation | 0.98 (0.88-1.09) | 0.71 | 1.00 (0.92-1.09) | 0.96 |

CI: confidence interval; ER: estrogen receptor; OR: odd ratio
